# Preparative Purification of Polyphenols from *Aronia melanocarpa* (Chokeberry) with Cellular Antioxidant and Antiproliferative Activity

**DOI:** 10.3390/molecules23010139

**Published:** 2018-01-10

**Authors:** Ningxuan Gao, Yuehua Wang, Xinyao Jiao, Shurui Chou, Enhui Li, Bin Li

**Affiliations:** Department of Food Science, College of Food, Shenyang Agriculture University, Shenyang 110161, China; gaoningxuan1990@163.com (N.G.); wangyuehua20122132@163.com (Y.W.); jiaoxinyaosyau@163.com (X.J.); choushurui@163.com (S.C.); 13940411312@163.com (E.L.)

**Keywords:** *Aronia melanocarpa*, polyphenols, purification, antioxidant, antiproliferative

## Abstract

The aim of this study was the purification process of polyphenols from *Aronia melanocarpa* (chokeberry), and the purification parameters were optimised by adsorption and desorption tests. By comparing adsorption and desorption ability of polyphenols from chokeberry on six kinds of macroporous resin, XAD-7 resin was selected. Experiments prove that the best purification parameters of static adsorption and desorption were sample pH = 4.0 with 4 h of adsorption; and desorption solvent is 95% ethanol (pH = 7.0) with 2 h of desorption. The best dynamic parameters were 9.3 bed volume (BV) of sample loading amount at a feeding flow rate of 2 BV/h, and washing the column with 5.8 BV of water, followed by subsequent elution with an eluent volume of 5.0 mL at an elution flow rate of 2 BV/h. Next the antioxidant and antiproliferative activity of polyphenols from chokeberry, blueberries, haskap berries was studied on HepG2 human liver cancer cells. The results show that polyphenol from chokeberry has a strong antioxidant effect. Taking into account the content of polyphenols in fruit, polyphenols from chokeberry represent a very valuable natural antioxidant source with antiproliferative products.

## 1. Introduction

*Aronia melanocarpa* (Rosaceae), also called chokeberry, is the small berry native to North America that has a high economic value. The flesh is deep red with a bitter and astringent taste. Chokeberry fruit is a dark blue and round berry. Generally, chokeberries is used for processing into fruit jams, fruit wines, fruit juices, dried fruit, canned-foods and other products.

Plant polyphenols are known for their unique antioxidant and antiproliferative activities. But there is a great difference between the components and the content of polyphenols in different kinds of plants. Due to its rich content of polyphenols, berries can be a good source of natural antioxidants [1,2,3]. In previous reports, it was confirmed that berry polyphenol substances can effectively remove free radicals from the body, reduce blood lipid levels and effectively regulate the body’s immune system, to maintain the normal function of the human body [4,5,6,7].

Early results showed that the fruit of *Aronia melanocarpa* (chokeberry) was rich in polyphenols. Reaching a total polyphenols content of 7.85 g/100 g (DW) [8]. The fresh red flesh also shows a large proportion of polyphenols in fruit pomace, but the bitter and astringent taste, it makes it hard for a lot for people to accept, but it can be used as a good source of natural antioxidants, to effectively utilize the rich polyphenol content of *Aronia melanocarpa* (chokeberry) [9,10].

The study shows that the free radicals produced during the oxidation of the body are strongly oxidizing and can damage tissues and cells in the body, which can lead to chronic diseases and aging effects [11]. Polyphenols, due to their redox properties, can be used as hydrogen donors, reductants, and singlet oxygen quenchers, thus playing a key anti-oxidation role [12]. Hwang found that, because it contains a lot of polyphenols, flavonoids and procyanidins, chokeberry extract showed stronger antioxidant activity than blueberry extract, allowing the body to reduce the damage from active oxygen free radicals [13]. In in vivo experiments, chokeberry extract also exhibited significant antioxidant activity in plasma and liver. Every 100 g of chokeberry fruit (dry weight) showed a DPPH free radical scavenging capacity of 279.38 μM Trolox equivalent. For ABTS free radical the scavenging capacity was 439.49 μM Trolox equivalent [8]. A thorough comparison of the anthocyanin content of nine kinds of blackberry, three kinds of raspberry, nine kinds of black currant, four kinds of red currant and chokeberry show that anthocyanin content of chokeberry was significantly higher than that of the other berries. The reasons for the high antioxidant activity of the extract were confirmed by comparing the antioxidant capacity of chokeberry fruit in different forms. It was found that the antioxidant capacity of fruit residue was significantly higher than that of fruit and fruit juice, which had a great relationship with the distribution and content of polyphenol in different parts of the fruit [8].

The hypolipidemic function of chokeberry has been widely praised in clinical research, and a study found that in rats fed a diet containing high levels of cholesterol, chokeberry juice can significantly hinder the increase of total cholesterol, low density lipoprotein and triglycerides in vivo, thus reducing the incidence of cardiovascular disease [6]. In addition, Kim et al.’s research demonstrated that the mechanism of the chokeberry extract reduction of cholesterol content is independent of the expression of genes related to cholesterol metabolism in the liver [14]. Due to the rich flavonoid content of the fruit, it can effectively inhibit pancreatic lipase, α-amylase and α-glucosidase so as to regulate the digestion and absorption of lipids and carbohydrates in the gastrointestinal tract, therefore chokeberry can be used as a raw material for functional foods, and the prevention of hyperglycemia and obesity, and the extract can be used for hyperglycemia and hyperlipemia treatment [15].

Macroporous resins are an effective method for the purification of plant polyphenols. They can greatly improve the purity of plant extracts. In this study, the purification of polyphenols by different types of macroporous resin was screened and the optimal static and dynamic adsorption−desorption conditions were obtained by optimizing the parameters of the purification process. On this basis, we also compared the antioxidant and antiproliferative activities of three kinds of berries (chokeberry, blueberries, haskap berries) using ‘in vitro’ assays.

## 2. Results

### 2.1. Purification of Polyphenols by Adsorption

#### 2.1.1. Screening of Suitable Adsorbents

**Figure 1:**
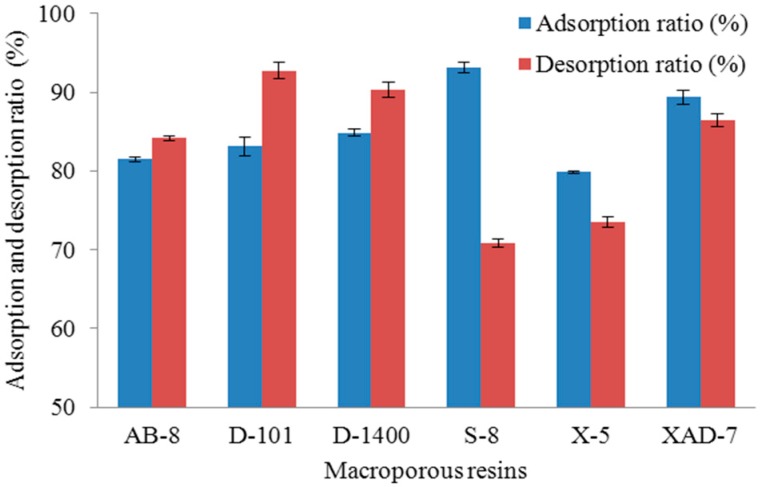
Adsorption and desorption ratio of different macroporous resins for chokeberry polyphenols.

Six macroporous resins with different adsorption capacity and desorption capacity were investigated for the separation and purification of chokeberry polyphenols. The results are presented in Figure 1. S-8 resin and XAD-7 resin showed a stronger adsorption capacity for chokeberry polyphenols than other adsorbents and D101 resin, D1400 resin and XAD-7 resin showed higher desorption ratios than the others, while S-8 resin showed the lowest desorption ratio and it can’t effectively desorb polyphenols. D101 resin and D1400 resin showed a lower adsorption capacity than XAD-7 resin, respectively.

On the basis of the stronger adsorption and desorption capacity for chokeberry polyphenols, XAD-7 resin was selected. Then, in order to make purification process more effective and efficient, the purification conditions needed to be optimised further.

#### 2.1.2. Static Adsorption and Desorption

**Figure 2:**
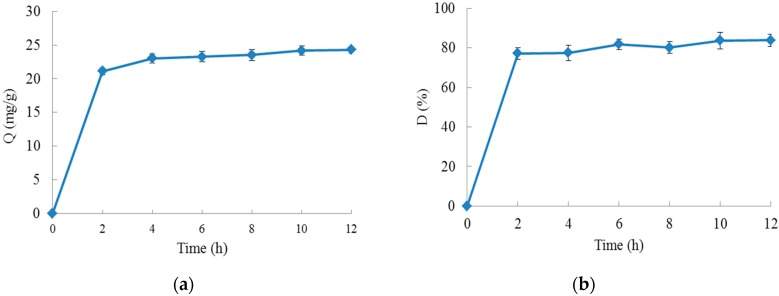
Adsorption and desorption properties of XAD-7 resin: (**a**) shows the static adsorption curve; (**b**) shows the static desorption curve. (Q (mg/g) is the adsorption capacity (mg GAE/g, gallic acid equivalents); D (%) is the desorption ratio.).

The adsorption and desorption kinetics on XAD-7 resins of chokeberry polyphenols are shown in Figure 2. As illustrated by the adsorption curve (Figure 2a), the chokeberry polyphenols were adsorbed rapidly by XAD-7 resins. During the first 2 h, the adsorption capacity increased rapidly, but then it increased slowly between 2 and 4 h. After 4 h, the adsorption capacity did not change significantly and equilibrium occurred, which suggested that the adsorption equilibrium occurred at around 4 h when the adsorption capacity was 23.02 ± 0.66 mg GAE/g. As shown by the desorption curve (Figure 2b), the chokeberry polyphenols absorbed into the resin were desorbed effectively by 70% (*v*/*v*) ethanol solution and the desorption ratio increased sharply within 2 h, separating the vast majority of chokeberry polyphenols from the XAD-7 resin, and solubilizing it in ethanol solution. After 2 h, when the desorption ratio was 77.14%, the desorption ratio did not show any further significant changes.

**Figure 3:**
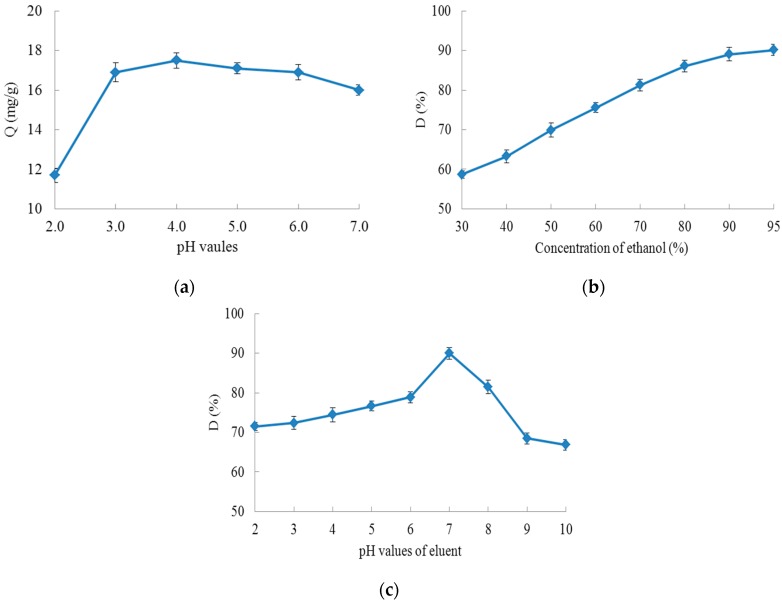
Static adsorption and desorption of XAD-7 resin: (**a**) is the effect of extract pH value on adsorption capacity; (**b**) is the effect of ethanol concentration on desorption ratio; (**c**) is the effect of eluent pH value on desorption ratio. (Q (mg/g) is the adsorption capacity; D (%) is the desorption ratio.).

Polyphenol molecules are slightly polar and acidic, and the form of the polyphenol molecules is affected by the pH value. Usually, they are easily adsorbed from lower pH value solutions by macroporous resins. Therefore the chokeberry polyphenol extract solution pH value greatly affects the adsorption capacity. As shown in Figure 3a, the adsorption capacity was increased with the rise of the chokeberry polyphenol extract solution pH value, when the pH value was less than 4.0, however, with further rise of the solution pH, the polyphenols exist as ions due to their ionisation reactions and the adsorption capacity of XAD-7 resin gradually decreased, so pH 4.0 was selected as the optimum pH value for the chokeberry polyphenol extract solution.

As shown in Figure 3b,c, the effect of elution can be effectively improved by adjusting the pH value or the ethanol concentration of the eluent. However a 100% desorption ratio of the adsorbed chokeberry polyphenols from the resin could not be achieved with any of the pH value or ethanol concentration. This was also visually confirmed from the residual purple color of the resin after the desorption experiments.

The ethanol concentration of the eluent is very important for the desorption effect of macroporous resins. As shown in Figure 3b, the desorption ratio of chokeberry polyphenols from XAD-7 resin obviously rose with increasing ethanol concentration, and reached a maximum value when the ethanol concentration was 95% (*v*/*v*). This phenomenon is caused by water which is more polar than ethanol. The polarity of the eluent was reduced with the increase in ethanol concentration. The XAD-7 is a slightly polar macroporous resin and the desorption ratio of chokeberry polyphenols increased as the polarity of the eluent and of the resin gradually became closer, therefore, 95% (*v*/*v*) was chosen as the optimum ethanol concentration of the eluent.

In order to know the effect of eluent pH value on the desorption ratio of chokeberry polyphenols from XAD-7 resin, studies were carried out with ethanol solution pH values from 2 to 10, and the results are summarized in Figure 3c. The dcesorption ratio increased as the pH value of the eluent rose from 2.0 to 7.0 and the highest desorption ratio was observed when the pH value of the eluent was 7.0. The desorption ratio obviously decreased as the pH value of the eluent rose, when the pH ranged between 7.0 and 10.0. Therefore, the optimum pH value of the elution was selected as 7.0.

#### 2.1.3. Dynamic Adsorption and Desorption

**Figure 4:**
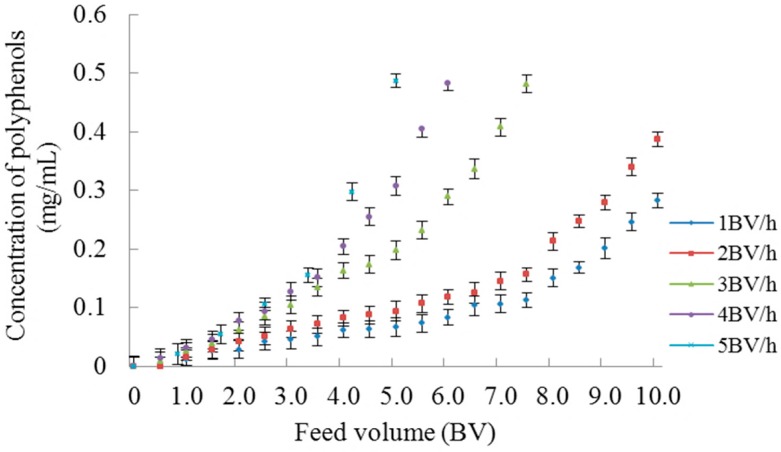
Dynamic breakthrough curves at different feed flow rates.

The dynamic breakthrough curves obtained on XAD-7 resin are based on the different feed flow rates and maximum feed volume. With the increase of the feed volume, adsorption would be reduced or even stopped, and as a result the polyphenols concentration in the effluent would gradually increase. Normally, the breakthrough point is used to determine the maximum feed volume, which can be defined as 10% of the ratio of the exit to the inlet solute concentration. The feed flow rate is an important factor in the dynamic adsorption that influences the maximum feed volume. As shown in Figure 4, polyphenol concentrations of the effluent rise with the increasing feed volume in the dynamic adsorption process. With the increase of the feed flow rate, the polyphenol concentration of the effluent increased in the same feed volume, leading to a decrease of the maximum feed volume. The chokeberry polyphenols start leaking out when feed volume reaches 4.0, 4.7, 5.8, 9.3 and 10.0 BV with the feed flow rates of 5, 4, 3, 2 and 1 BV/h, which are their breakthrough points, respectively. There is insufficient time for the polyphenol molecules to be adsorbed on the macroporous resin at the higher feed flow rates, but a lower feed flow rate would extend the experimental cycle. Therefore, 2 BV/h was selected as the optimal feed flow rate because the feed volume did not show large differences between a flow rate of 1 BV/h and 2 BV/h and the production cycle was too long when the flow rate was lower than 2 BV/h.

**Figure 5:**
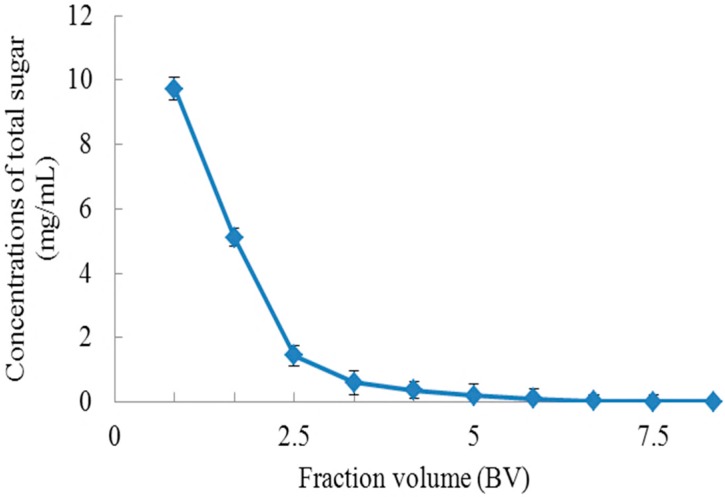
Dynamic water flushing curve.

As the chromatography column contains a large amount of non-adsorbed polyphenols and saccharides, it was rinsed with deionized water to remove the impurities. As shown in Figure 5, with the increase of the amount of deionized water elution, the concentrations of total sugar in the effluent decreased significantly. When the amount of deionized water used reached 4.2 BV, the effluent became colorless, indicating that non-adsorbed products had been effectively removed.

It can be seen from Figure 5 that approximately 5.8 BV deionized water could completely elute the total sugar. Hence, 5.8 BV of deionized water is inferred as the most suitable conditions for removing the impurities.

**Figure 6:**
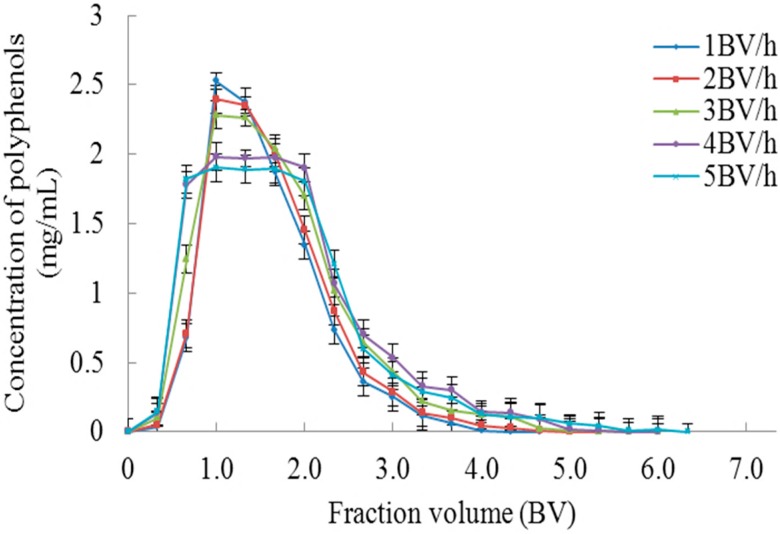
Dynamic desorption curves at different elution flow rates.

The dynamic desorption curve was drawn based on the volume of eluent and the concentration of polyphenols therein at different flow rates. Ethanol (55%, *v*/*v*) was used for elution of polyphenols by maintaining the flow rates at 1, 2, 3, 4 and 5 BV/h. As shown in Figure 6, as the eluent flow rate increases, the dynamic desorption curve peak broadens, and the tailing phenomenon becomes more obvious, resulting in increased elution volume. Hence a lower elution flow rate is conducive to elution of chokeberry polyphenols but the elution flow rate is too slow, so the elution time will be increased accordingly. Therefore, 2 BV/h was selected as the optimal elution flow rate. It can be seen from the figure that approximately 5 BV eluent could completely elute the chokeberry polyphenols from the XAD-7 resin at a flow rate of 2 BV/h.

### 2.2. Cellular Antioxidant Activity 

In order to better understand the antioxidant ability of the purified chokeberry polyphenol products and to compare the antioxidant properties of polyphenols from different sources (chokeberry, blueberry and haskap) cellular antioxidant activity tests (CAA) with HepG2 cells were performed. It can be seen from Table 1 that the antioxidant activity of polyphenols from chokeberry was slightly lower than for blueberry (the corresponding CAA values were 334.48 and 357.64 umol Q/100 ug, respectively), and haskap showed the lowest CAA value (252.87 umol Q/100 ug). This may be due to differences in the polyphenol components of the different sources.

### 2.3. Antiproliferation Activity and Cytotoxicity 

**Figure 7:**
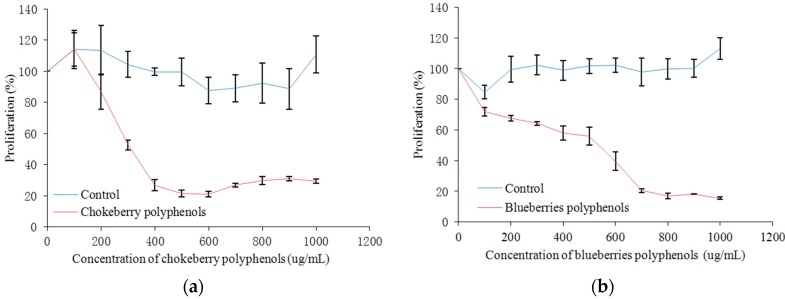
Inhibition of HepG2 cell proliferation by polyphenols from chokeberry (**a**); blueberry (**b**); haskap (**c**) berries.

**Figure 8:**
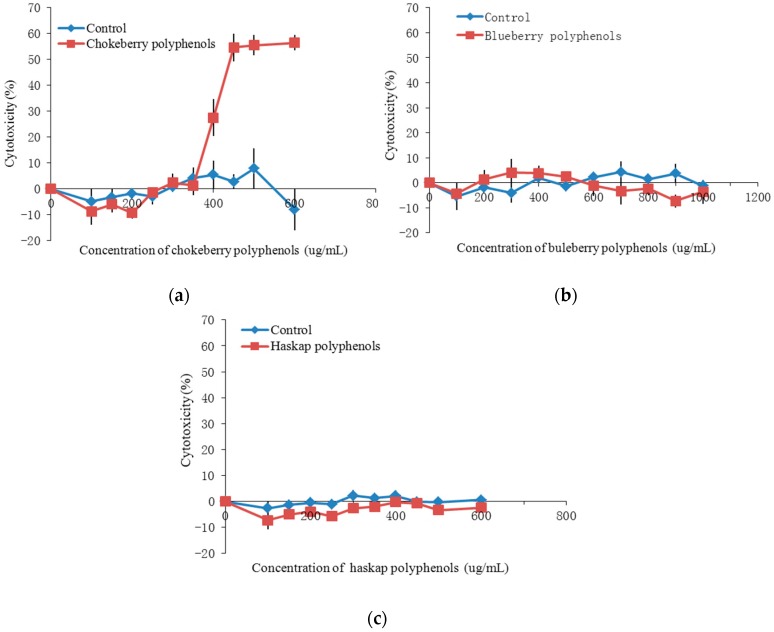
Cytotoxicity of polyphenols from chokeberry (**a**); blueberry (**b**); haskap (**c**) berries.

The inhibition of HepG2 human liver cancer cell proliferation in vitro by the polyphenols from chokeberry, blueberries, haskap berries and their cytotoxic effects are presented in Table 1 and Figure 7 and Figure 8. In general, these berry polyphenols have potent antiproliferation activity, inhibiting HepG2 cell growth in a dose dependent manner. By comparing the corresponding IC_50_ values it can be found that the chokeberry and blueberry polyphenols had higher inhibitory effects on HepG2 proliferation. The antiproliferative activity of chokeberry polyphenols (IC_50_ = 338.36 ug/mL) was slightly higher than that of polyphenols from blueberries, but much higher than the haskap berries. When the concentration was less than 350 ug/mL chokeberry polyphenols showed no cytotoxic effects, which indicated that the inhibition of proliferation was not caused by cytotoxicity.

## 3. Discussion

Chokeberry fruit is rich in polyphenols and the antioxidant activity of its extracted compounds is several times higher than for other berry extracts. Wojdylo et al. [8], found that the total phenolic content of chokeberry fruit reached 7849.21 mg/100 g (dry weight), which is mainly composed of procyanidins, which account for 66% of the total phenol content. The anthocyanin content accounted for 25% of the total phenol content, and consists of four components, namely cyanidin 3-*O*-galactoside, 3-*O*-glucoside, 3-*O*-arabinoside and 3-*O*-xyloside, of which the 3-*O*-galactoside id the main one. The content of chlorogenic and neochlorogenic acid accounts for 7.5% of the total phenol content, and the phenolic acid content in fruit juice is far greater than the phenolic acid content of fruit and fruit residue due to its good water solubility. Compared with the anthocyanins and the proanthocyanins, the flavonoid content is lower, at about 1.3% of the total phenolic content. Wojdylo et al. identified three types: quercetin 3-rutinoside, quercetin 3-galactoside and quercetin 3-glucoside, respectively. In addition, Slimestad et al. [16] used 1D- and 2D-nuclear magnetic resonance experiments together with electrospray mass spectrometry analysis of chokeberry fruit and identified eriodictyol 7-*O*-β-glucuronide, together with the rare flavonols quercetin 3-vicianoside, quercetin 3-robinobioside and other quercetin glycosides and they determined the flavonoids content of fresh chokeberry fruit. It was found that each 100 g of fruit contains > 71 mg of flavonoids. In addition, after comparison, it was found that the polyphenol content of chokeberry fruit pomace was higher than that of fruit and fruit juice.

The extraction process of chokeberry extract used in this study was the result of previous research at the authors’ laboratory. This extraction process can effectively extract polyphenols of chokeberry and an extract yield of 19.549 mg/g. In a study on antioxidant active substance extraction by Grunovaite et al. [9], using different extraction solvents (acetone, ethanol, ethane, water and supercritical fluid extraction-CO_2_), it was found that chokeberry fruit residue was a good source of antioxidants. The results show that the antioxidant components of chokeberry in more easily soluble in ethanol solution, which helps to extract antioxidant substances of chokeberry more thoroughly.

The purification process parameters of polyphenols from chokeberry in this study have been validated and put into production in the factory of Shenyang Huangguan Blueberry Biotechnology Co., Ltd. (Shenyang, China). At present, the purification of polyphenols from plants is mainly through macroporous resins. The types of macroporous resin mainly selected for purification of polyphenols are AB-8, D141, XAD-7 and X-5. Sun et al. [17], selected X-5 resin by comparing eight kinds of macroporous resin and successfully purified polyphenols from apple using this resin. The concentration of polyphenols increased from 35.17% to 74.64%. Previous studies have found that the XAD-7 resin has a better effect on anthocyanins in purple potatoes and jamun [18,19].

The hypolipidemic function of chokeberry has been widely highlighted in clinical research. Worsztynowicz et al., through determination of the activity of porcine pancreatic lipase and amylase experiments further found that chokeberry polyphenols can effectively inhibit the activity of pancreatic lipase and α-amylase [20] but the different polyphenol components have very different degrees of inhibition of the various digestive enzymes. The main inhibition effect on pancreatic lipase and α-amylase, respectively, is due to the phenolic acids and anthocyanins in the chokeberry polyphenol composition. Through further experiments, it was found that cyanidin 3-*O*-glucoside was the most effective inhibitor of pancreatic lipase (IC_50_ = 1.74 ± 0.04 mg/mL) and α-amylase was inhibited the strongest by chlorogenic acid (IC_50_ = 0.57 ± 0.16 mg/mL). In addition, cyanidin 3-*O*-glucoside can also greatly inhibit the reactions catalyzed by pancreatic lipase (IC_50_ = 1.17 ± 0.05 mg/mL).

Using mice to test chokeberry polyphenolic compounds in vivo, it was found that chokeberry polyphenols have potential immunoregulatory and anti-inflammatory functions [21]. The complement system is an important component of the innate immune system, and it includes a set of serum proteins and membrane receptors mainly involved in the dissolution of foreign cells, inflammation and phagocytosis process, and thus play a role in resistance to infection. It is found that the procyanidins C1, B5 and B2 and various aglycones of cyanidin have strong complement-fixing activities and the activity of the cyanidin 3-glucoside was stronger than that of the other anthocyanins. The excessive production of nitric oxide (NO) by macrophages could promote the development of rheumatoid arthritis, septic shock and other autoimmune disorders, so the inhibition of NO has potential therapeutic value for inflammatory diseases. Giang et al.’s research work found that the procyanidins C1, B5 and B2 and proanthocyanidin-rich fractions could show inhibitory activities on nitric oxide (NO) production in LPS-stimulated RAW 264.7 mouse macrophages.

Regarding the anti-inflammatory function, Martin found that chokeberry polyphenols could inhibit the spleen cells C57/BL6 of mice to produce IL-6 by LPS induction [22]. IL-6 is associated with autoimmune diseases, such as multiple sclerosis, arthritis and enteritis. This further proves the positive anti-inflammatory role of chokeberry components.

Fibrinogen, also called coagulation factor I, accounts for 4% of the total human plasma protein. It mainly involved in the last stage of the blood coagulation cascade. However, the fibrinogen is especially sensitive to compounds like peroxynitrite with strong oxidative and nitrative ability [23]. Reactions between fibrinogen and peroxynitrite will cause both structural modifications and changes of the biological properties of plasma glycoprotein [24]. The study found that chokeberry extract (hydroxycinnamic acids, anthocyanins and flavanols ((+)-catechin, (−)-epicatechin and procyanidins) could obviously inhibit the nitration of fibrinogen and high molecular weight protein aggregates induced by the peroxynitrite. That black chokeberry extracts can effectively protect the plasma fibrinogen to avoid peroxynitrite-induced nitrative damage, and play a protective role in peroxynitrite-related cardiovascular diseases [25].

## 4. Materials and Methods 

### 4.1. Materials

Chokeberry fruits were harvested by hand in Zhuanghe, Liaoning Province, China, in June 2015 and were authenticated by the Liaoning Fukangyuan Aroniamelanocarpa Technology Development Co., Ltd., Haicheng, China. The fruits were washed thoroughly with deionized water, followed by placement in polyethylene bags and storage at −20 °C prior to extraction. The plant was further classified at the Food Science Laboratory of the College of Food, Shenyang Agriculture University, Shenyang, China, as chokeberry.

Blueberry and haskap berry fruits were picked in Liaoning Province of China. Blueberry and haskap polyphenols were prepared by the Food Science Laboratory of the College of Food, Shenyang Agriculture University.

### 4.2. Extraction of Polyphenols from Chokeberry

Chokeberry was thawed at room temperature, pulped and and then the pulp was conserved for further use. In our previous study [26], the polyphenols of chokeberry (10 g) were extracted with 55% (*v*/*v*) ethanol-water solution (440 mL) for 90 min at 45 °C with ultrasonic assistance. After extraction, the polyphenol extract were filtered using a Buchner funnel and Whatman No. 2 filter paper under vacuum. The supernatants were concentrated to remove ethanol with a rotary evaporator under reduced pressure at 45 °C. To obtain chokeberry polyphenols extract the crude polyphenols aqueous solution is used.

### 4.3. Determination of Total Polyphenols

The total polyphenols contents (TPC) was determined by the colorimetric Folin-Ciocalteu method with some modificatins [27]. Using gallic acid as standard, and after drawing a standard curve, the TPC was determined as milligram gallic acid equivalents per 1 mL (mg GAE/mL).

### 4.4. Purification of Polyphenols by Adsorption

#### 4.4.1. Adsorbents Preparation

Six adsorbents were used for purification experiments: AB-8, D-101, D-1400, S-8, X-5, XAD-7 macroporous resins. Prior to use, the polymeric adsorbents were activated by soaking with 95% ethanol for 24 h and subsequently washing thoroughly with deionized water. Then the polymeric adsorbents were soaked with 5% HCl and 2% NaOH solutions successively for 12 h, and the resins were thoroughly washed with deionized water until they became neutral.

#### 4.4.2. Screening of Suitable Adsorbents

Adsorption equilibrium experiments were carried out at room temperature to compare among the six adsorbents. 5 g of each pretreated adsorbent was mixed with 50 mL of ethanol-free chokeberry polyphenols extract in a 100 mL Erlenmeyer flask. The flask was placed in a shaking incubator so as to maintain the agitation at 120 rpm and constant temperature for 24 h to reach adsorption equilibrium. The resulting liquid phase was separated from the adsorbent by filtration under vacuum, and the total polyphenols concentration in the liquid phase was determined. The adsorption capacity of polyphenols was calculated with Equation (1); and the adsorption ratio was calculated with Equation (2):

(1) $$Q=\frac{(C_{0}-C_{1})\times V_{1}}{M},$$

(2) $$E\;(\%)=\frac{C_{0}-C_{1}}{C_{0}}\times 100,$$

where Q (mg/g) is the adsorption capacity; C_0_ (mg/mL) is the initial concentration of polyphenols in the polyphenols extract solution from chokeberry; C_1_ is the equilibrium concentration of polyphenols; V_1_ (mL) is the volume of polyphenol extract solution used; M (g) is the mass of adsorbent; E (%) is the adsorption ratio;

After adsorption equilibrium was reached, the adsorbent was washed using deionized water to remove excess polyphenols. Then it was desorbed with 50 mL of 70% ethanol solution in the 100 mL Erlenmeyer flask. The flask was shaken on a shaking incubator at a constant temperature for 24 h. The total polyphenols concentration in the eluent solution was determined. The desorption ratio was calculated with Equation (3):

(3) $$D\;(\%)=\frac{V_{2}\times C_{2}}{M\times Q}\times 100,$$

where D (%) is the desorption ratio;V_2_ (mL) is the volume of eluent solution; C_2_ is the concentration of the polyphenols in the eluent solution; Q and M are the same as those defined above. All experiments were replicated three times.

#### 4.4.3. Static Adsorption and Desorption Tests

The properties of the six resins were evaluated by their adsorption capacity, adsorption ratios and desorption ratios. The adsorption property of XAD-7 resin selected from the preliminary experiments was investigated in further experiments.

The ethanol-free chokeberry polyphenols extract was adjusted to different pH values using 1.0 mol/L HCl and 1.0 mol/L NaOH solutions. Then 50 mL of each solution was adsorbed by 5.0 g XAD-7 resin as described above and the total polyphenols concentration in the liquid phase was determined when adsorption equilibrium was reached. The adsorption capacities were calculated in order to study the relationship between the pH value of the polyphenols extract and the adsorption capacities of XAD-7 resin.

After adsorption equilibrium was reached, the XAD-7 resin was washed using deionized water to remove excess chokeberry polyphenols. Each of the 5.0 g samples of XAD-7 resin was desorbed with 50 mL ethanol solutions of different concentration and the total polyphenols concentration in the eluent solutions was determined. The desorption ratios at different concentrations of ethanol solution were calculated in order to investigate the effects of different ethanol concentrations in the desorption capacities of XAD-7 resin. The ethanol solution was adjusted to a different pH value using 1.0 mol/L HCl and 1.0 mol/L NaOH solutions. Each 5.0 g sample XAD-7 resin was desorbed with 50 mL ethanol solution of different concentration as described above and the total polyphenols concentration in the eluent solution was determined. The desorption ratio was calculated in order to investigate the relationship between desorption ratio and different eluent solution pH value.

#### 4.4.4. Dynamic Adsorption and Desorption Tests

Dynamic adsorption and desorption experiments were carried out on a glass column (18 × 300 mm) wet-packed with pretreated XAD-7 macroporous resin at room temperature, and the bed volume (BV) of the resin was 60 mL. Then the ethanol-free chokeberry polyphenols extract (3.0 mg/mL) were passed through the glass column at different flow rates. The effluent solution was collected with a fraction collector. The concentration of the total polyphenols of the effluent solution was determined and the adsorptive saturation was analyzed in order to investigate the relationship between adsorption capacities and feed flow rates. Washing of the loaded resin was done with deionized water (2 BV/h) to remove the non-adsorbed polyphenols, saccharides, proteins and unknown impurities from the chromatography column. The effluent solution was collected with the fraction collector. The concentration of the total sugar of the effluent solution was determined with the anthrone colorimetric method in order to investigate the relationship between effect of impurit δ removal and the amount of deionized water used.

Desorption of polyphenols from the loaded XAD-7 macroporous resin was performed using aqueous ethanol solutions (55% *v*/*v*). Washing of the loaded resin was done with deionized water prior to desorption. The ethanol solution was passed through the glass column at different flow rates. The effluent solution were collected with a fraction collector. The concentration of the total polyphenols of the effluent solution was determined and usage amount of ethanol solution was recorded at different flow rates in order to investigate the relationship between effect of desorption and flow rates. At the same time, usage amount of ethanol solution was determined at the optimum flow rate.

### 4.5. Cytotoxicity Measurement

The cytotoxicity of polyphenols from chokeberry, blueberries, haskap berries toward HepG2 cells was measured using a method previously described by the Liu lab [28]. Briefly, cells were placed in growth medium at a concentration of 4.0 × 10^4^/well in a 96-well flat-bottom plate. To sufficiently attach cells, they were incubated for 24 h at 37 °C in 5% CO_2_. After removing the medium, cells were washed with 100 μL sterile cold PBS once. Then 100 μL of medium with different concentrations of polyphenols from chokeberry, blueberries and haskap berries were added to each well; the treatment medium without polyphenols added to the well served as the control. The treatment medium was removed and the cells were washed with PBS, after 24 h of incubation at 37 °C. The cytotoxicity was determined by the methylene blue assay, whereby 50 μL of methylene blue solution (98% HBSS, 0.67% glutaraldehyde, and 0.6% methylene blue) was added to the each well of the culture plate, and incubated at 37 °C. After 60 min, plate was rinsed using water until the water became clear. Then, 100 μL of elution buffer (49% PBS, 50% ethanol, and 1% acetic acid) was added to each well with a multichannel pipette and it was place on a plate rotator at room temperature for 30 min. Elution solutions were centrifuged and transferred to a 96-well plate. The absorbance was read with a microplate reader at 570 nm wavelength. Cytotoxicity was considered for different concentrations of polyphenols by a 10% reduction in the absorbance compared to the control.

### 4.6. Cellular Antioxidant Activity

The CAA of polyphenols from chokeberry, blueberries, haskap berries were determined using the Liu lab method [29]. Briefly, human hepatocellular carcinoma HepG2 cells were seeded at a density of 6 × 10^4^/well on a 96-well plate in 100 μL of growth medium/well. Twenty-four hours after seeding, the growth medium was removed, and the wells were washed with 100 μL PBS once. Wells were treated in triplicate for 1 h with 100 μL of solutions containing different concentration chokeberry polyphenols plus 25 μM DCFH-DA at 37 °C. Then, to remove treatment medium, cells were washed with 100 μL PBS once. Next the 100 μL HBSS containing no ABAP was added to the blank wells and 100 μL of 600 μM ABAP in HBSS to other wells. The 96-well plate was placed into a Fluoroskan Ascent FL plate-reader (Thermo Fisher Scientific, Waltham, MA, USA) at 37 °C immediately. The emission wavelength at 538 nm was measured after an excitation at 485 nm every 5 min for 1 h. On each 96-well plate include control and blank wells. Blank wells contain cells treated with DCFH-DA and no ABAP, control wells contain cells treated with DCFH-DA and ABAP. After blank subtraction from the initial fluorescence values, the area under the curve of fluorescence versus time was integrated to calculate the CAA value at each concentration of chokeberry polyphenols as follows:

CAA unit = 100 − (∫SA/∫CA) × 100,(4)

where ∫SA is the integrated area under the sample fluorescence versus time curve and ∫CA is the integrated area from the control curve. The median effective dose (EC_50_) was determined for the chokeberry polyphenols from the median effect plot of log (*f*a/*f*u) versus log (dose), where *f*a is the fraction affected (CAA unit) and *f*u is the fraction unaffected (1-CAA unit) by the treatment. The EC_50_ values are given as the mean ± SD for triplicate sets of data obtained from the same experiment. EC_50_ values were converted to CAA values, which are expressed as micromoles of quercetin equivalents (QE) per 100 μmol of chokeberry polyphenols.

### 4.7. Cellular of Antiproliferative Activity

The antiproliferative activity of polyphenols from chokeberry, blueberries, haskap berries were determined using a previously reported method from the Liu lab [30]. Briefly, HepG2 cells at concentrations of 2.5 × 10^4^/well in the growth media were placed in each well of a 96-well flat-bottom plate and were incubated at 37 °C in 5% CO_2_ to allow cells to attach sufficiently. Then the growth medium was removed, and the cells were washed once with 100 μL sterile cold PBS. Afterwards, the HepG2 cells were treated with various concentrations of purified polyphenols (chokeberry, blueberries and haskap) in growth medium. Control cultures received the polyphenols solution minus the polyphenols and blank wells contained 100 μL of growth medium without HepG2 cells. At least three replicates were performed for each sample. Then the cells were incubated at 37 °C in 5% CO_2_ again for 72 h. The HepG2 cells were stained and assayed by the methylene blue method. Absorbance was measured at 570 nm using a microplate reader. The percentage of cell proliferation was calculated by absorbance of each concentration polyphenols compared to the control. The antiproliferative activity was expressed as an IC_50_ value.

## 5. Conclusions

In this study, the adsorption and desorption properties of six kinds of macroporous resins for chokeberry polyphenols were determined, and the purification processing parameters were optimised. XAD-7 macroporous resin was selected as the best purification material. The best purification parameters of static adsorption and desorption were pH of 4 for the sample solution for 4 h of adsorption, and desorption solvent was 95% ethanol (pH = 7.0) with 2 h of desorption; the best purification parameters of dynamic adsorption and desorption were 9.3 BV of sample loading amount at a feed flow rate of 2 BV/h, and washing the column with 5.8 BV of water, followed by subsequent elution with an eluent volume of 5.0 BV at flow rate for elution of 2 BV/h. Under the purification conditions, the purity of chokeberry polyphenols increased from 11.62% to 64.37%.

On the basis of the above, contrasting antioxidant and antiproliferative activities of three kinds of berry (chokeberry, blueberries, haskap berries) polyphenols by HepG2 human liver cancer cells, the results show that chokeberry polyphenols have a stronger antiproliferative activity than the other kinds of berry polyphenols. In vitro antioxidant analysis of chokeberry polyphenols on HepG2 cells showed high antioxidant activity, and the CAA value is 334.48 umol Q/100 ug, only slightly lower than that of blueberry polyphenols. Chokeberry fruit can therefore be considered a good source of natural antioxidants because of its polyphenol content.

## Figures and Tables

**Table 1:** CAA, EC_50_ and antiproliferative activities (IC_50_) of polyphenols from the three kinds of berries toward HepG2 cells.

	CAA (umol Q/100 ug)	EC_50_ (ug/mL)	IC_50_ (ug/mL)
Chokeberry	334.48 ± 29.57	0.069 ± 0.005	338.36 ± 23.17
Blueberry	357.64 ± 33.61	0.064 ± 0.006	359.74 ± 29.38
Haskap	252.87 ± 23.15	0.049 ± 0.003	552.07 ± 34.19
